# A Nanostructure with Defect Based on Fano Resonance for Application on Refractive-Index and Temperature Sensing

**DOI:** 10.3390/s20154125

**Published:** 2020-07-24

**Authors:** Xiaoyu Yang, Ertian Hua, Hao Su, Jing Guo, Shubin Yan

**Affiliations:** 1 School of Instrument and Electronics, North University of China, Taiyuan 030051, China; 18734146697@163.com (X.Y.); suhao226401@163.com (H.S.); 2 School of Electrical Engineering, Zhejiang University of Water Resources and Electric Power, Key Laboratory for Technology in Rural Water Management of Zhejiang Province, Hangzhou 310018, China; het@zjut.edu.cn (E.H.); guojing@zjweu.edu.cn (J.G.)

**Keywords:** metal-insulator-metal, Fano resonance, refractive-index nanosensor, temperature sensing

## Abstract

Herein, a nanosensor structure is proposed, which comprises metal-insulator-metal (MIM) waveguide with stub and circular ring cavity with a stub (CRCS). The phenomenon of Fano resonance appears in the transmission spectrum, which is formed by interaction between the narrowband mode of CRCS and broadband mode of stub on bus waveguide. The influence of geometric asymmetry on mode splitting of Fano resonance was discussed. The mode splitting of Fano resonance can vastly improve figure of merit (FOM) with a sight decrease of sensitivity. The best performance of the refractive-index nanosensor is attained, which is 1420 nm/RIU with a high FOM of 76.76. Additionally, the application of designed structure on temperature sensing was investigated, which has sensitivity of 0.8 nm/°C. The proposed structure also possesses potential applications on other on-chip nanosensors.

## 1. Introduction

Surface plasmon polaritons (SPPs) are charge-density waves, which greatly decay in the direction vertical to the metal-dielectric interface so that they are largely trapped on the interface [1,2]. Thus, SPPs have many good properties, such as getting over the classical diffraction of light limit and controlling light within nanoscale [3,4,5]. Various optical devices based on SPPs were extensively reported, including filters [6,7,8,9], optical switching [10,11], splitters [12,13], nanosensors [14,15,16,17,18], and couplers [19]. Among these, the SPPs-based nanosensor is a significant application, which has advantages of smaller size as well as being easy to integrate into optical circuits and get in touch with sensing mediums. In addition, Fano resonance [20,21,22,23,24,25] is observed in plasmon coupled structures, which is generated by the interference between narrowband mode and broadband mode. According to previous works, making proper defects on waveguide or resonator cavity has a big chance to form Fano resonance. Additionally, different shapes and positions of defects induce diverse Fano resonance. Wu et al. [26] added a triangle defect on the waveguide to induce the phenomenon of Fano resonance with a sharp dip. Chen et al. [27] made a baffle on the waveguide, which leads to the formation of Fano resonance with a sharp peak. As for defects on the resonator, lots of works made splits on that [16]. However, less work added a stub on the resonator to form Fano resonance, which is studied in this paper. Fano resonance with an asymmetric sharp outline is easy to be distinguished, so it displays comparatively narrow full width at half maximum (FWHM) [28], high sensitivity, and sharply spectral response. FWHM can represent the spectral width of Fano resonance, and narrower FWHM is the reason for better optical resolution and figure of merit (FOM). Besides, wavelength of Fano resonance is easy to be adjusted by changing geometry structures or sensing mediums.

Metal-insulator-metal (MIM) waveguides, a kind of SPPs-based waveguides, have remarkable properties, for instance, easy manufacture, smaller dimension, stronger localized field confinement, shorter transmission length, and low propagation loss [29,30,31]. So far, plenty of sensors based on MIM waveguide and Fano resonance have been widely discussed. Yan et al. [16] presented coupled structure of notched ring cavity and MIM waveguide with stub, whose sensitivity is 1071.4 nm/RIU with an FOM of 14.29. Wang et al. [14] designed a nanosensor with sensitivity of 680 nm/RIU and FOM of 8.68, which comprises an analogy T shape cavity and MIM waveguide. Kong et al. [32] proposed a temperature sensor consisting of stub coupled with rectangle resonator, which could obtain a sensitivity of 0.36 nm/°C. In this paper, when the proposed structure serves as a refractive index sensor, its sensitivity can reach 1420 nm/RIU with an FOM of 76.76. When the proposed structure acts as a temperature sensor, its sensitivity can reach 0.8 nm/°C.

In this work, a nanosensor structure is proposed, which comprises a MIM bus waveguide with stub and a circular ring cavity with a stub (CRCS). The propagation characteristics were demonstrated by the finite element method (FEM). The previous papers only concentrated on studying the effects of structural parameters on existed Fano resonance. Here, however, the influence of geometric asymmetry on mode splitting of Fano resonance was investigated by analyzing the symmetric structures and asymmetric structures, which provides a new way to improve the FOM with slight decrease of sensitivity. The effects of structural parameters on the position and spectral width of Fano resonances were discussed. Additionally, the applications of designed structure on refractive-index sensing and temperature sensing were studied in detail. Furthermore, the designed structure can detect any physical quantity that is dependent on refractive index. For instance, when the blood samples fill into the CRCS, the designed structure can act as a biosensor to detect the concentration of the hemoglobin level in the blood group.

## 2. Geometry Model and Analysis Method

**Figure 1:**
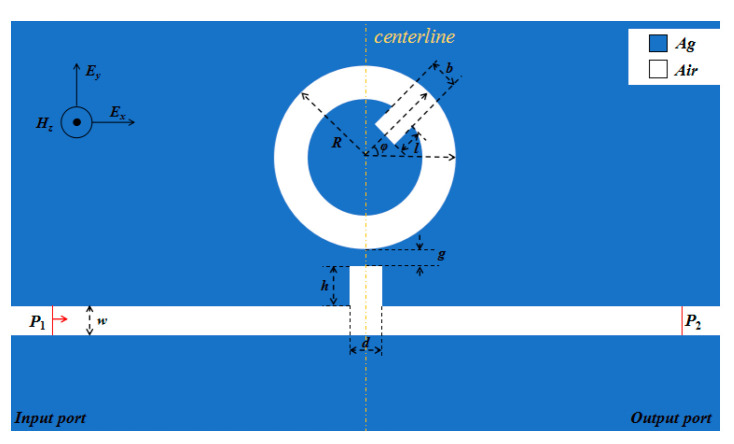
2D schematic of the designed structure.

The COMSOL Multiphysics 5.4a is used to construct the geometry model. The z dimension of metal is very larger than the light wavelength. Therefore, the 2D model, employed to illustrate the structure, can approximately replace the 3D model with extremely slight departure. The absorbing boundary condition in all boundaries is acted by the perfect matched layer. Additionally, ultra-fine meshing is selected to guarantee the precision of the simulation. As plotted in Figure 1, the designed structure is composed of a bus waveguide with stub and a circular ring cavity with a stub (CRCS). Except the stub on the CRCS, the structure is symmetric about the centerline. The outer radius of the CRCS is denoted by *R*. The length and width of the stub on CRCS are *l* and *b*, respectively. The angle between horizontal axis and centerline of stub on CRCS is *φ*. The coupling gap is *g*. The height and width of the stub on bus waveguide are *h* and *d*, respectively. The width of bus waveguide and the circular ring cavity maintains constant (i.e., *w* = 50 nm). Thus, the coupled system only excites fundamental transverse magnetic (TM) mode, and its equation in the MIM waveguides is as follow [33,34]:

tanh (*kw*) = −2*kpα*_c_/(*k*^2^ + *p*^2^*α*_c_^2^), (1)

where *k* is wave vector in the waveguide, *p* = *ε*_in_/*ε*_m_, *α*_c_ = [*k*_0_^2^ (*ε*_in_ − *ε*_m_) + *k*]^1/2^. The *ε*_in_ and *ε*_m_ are permittivity of dielectric and metal, respectively. The *k*_0_ = 2*π*/*λ*_0_ is the wave vector in free space.

The silver is chosen to be the filled metal. Because silver has lower power-consumption, in other words, the electromagnetic response of silver has a relatively small imaginary part of relative permittivity to ensure a relatively strong electric field intensity in the waveguide and resonator. The silver and air are denoted by blue and white districts in Figure 1, respectively. The proposed structure can be fabricated by the focused ion beam (FIB) method, which is that the microstructure on the silver film deposited on quartz substrate can be formed by sputtering the focused ion beam. The SPPs only can be supported in the interface between metal and dielectric rather than the interface of two dielectrics. Thus, the SPPs only can propagate in the waveguide and ring cavity, which are on the surface of the substrate quartz. P_1_ and P_2_ respectively denote input port and output port. The light gradually couples into the single mode fiber and grating to generate an electromagnetic wave at P_1_. Any light source that can couple into single mode fiber can serve as the interrogation device. Especially, laser is always chosen to be the light source, and the ordinary light source is hard to meet the requirement. Then, SPPs could be excited at P_1_, and then it would propagate into the bus waveguide. When phase-match condition is satisfied, the SPPs could couple into the CRCS to form resonance. The phase-match condition is:

∆*θ* = 2*πm*, (2)

where mode order is *m* = 2*L*/*λ*_spp_, *L* is effective length of resonator, wavelength of SPPs is *λ*_spp_ = *λ*_0_/Re(*n*_eff_), real part of effective refractive-index is Re(*n*_eff_) = [*ε*_m_ + (*k*/*k*_0_)^2^]^1/2^, *k* can be obtained by Equation (1).

Sensitivity (S) and figure of merit (FOM) are two considerable parameters to judge performance of sensor, which are calculated as follows [35]:

S = ∆*λ*/∆*n*, (3)

FOM = S/FWHM,(4)

where ∆*λ* is change of resonance wavelength and ∆*n* is variation of refractive indices.

## 3. Simulations and Results

**Figure 2:**
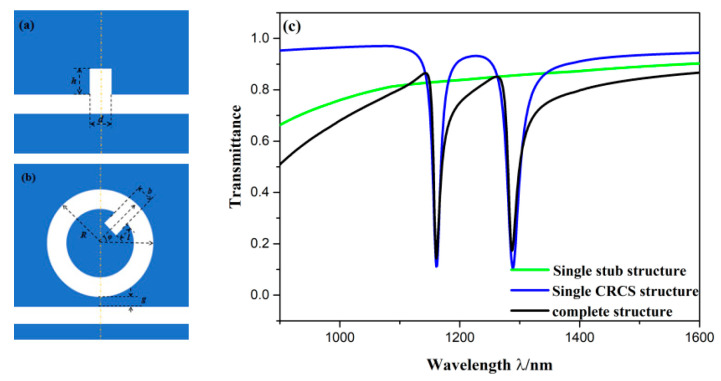
2D schematic of (**a**) single stub structure; (**b**) single CRCS structure; (**c**) Transmission spectra of the single stub structure (green line), single CRCS structure (blue line), and complete structure (black line).

To understand the superiority of the presented sensor structure that is derived from the single CRCS structure, single stub structure, single CRCS structure, and complete structure were discussed. The single stub structure includes a bus waveguide and a stub on waveguide, which is shown in Figure 2a. The single CRCS structure consists of a bus waveguide couple with CRCS, which is plotted in Figure 2b. Their transmission spectra are depicted in Figure 2c, which are denoted by green, blue, and black curves, respectively. The three structures have the same geometric parameters, which are as follows: *R* = 160 nm, *b* = *d* = 50 nm, *l* = *h* = 80 nm, *φ* = 45°, *g* = 10 nm. It is found that the green curve had an almost horizontal outline, whose transmittance was always in the range from 0.63 to 0.85. In other words, the width of its spectrum was very wide so that it could be treated as the broadband mode. The spectrum of single CRCS structure had two dips, at which transmittance was as low as 0.1 and the spectral width was very narrow. Thus, it could be treated as the narrowband mode. The spectrum of complete structure had two dips and possessed an asymmetric profile, which indicates the emergence of Fano resonance. The complete structure is composed of single stub structure and single CRCS structure. It can be concluded that Fano resonance is formed from the interference of the broadband mode and narrowband mode.

**Figure 3:**
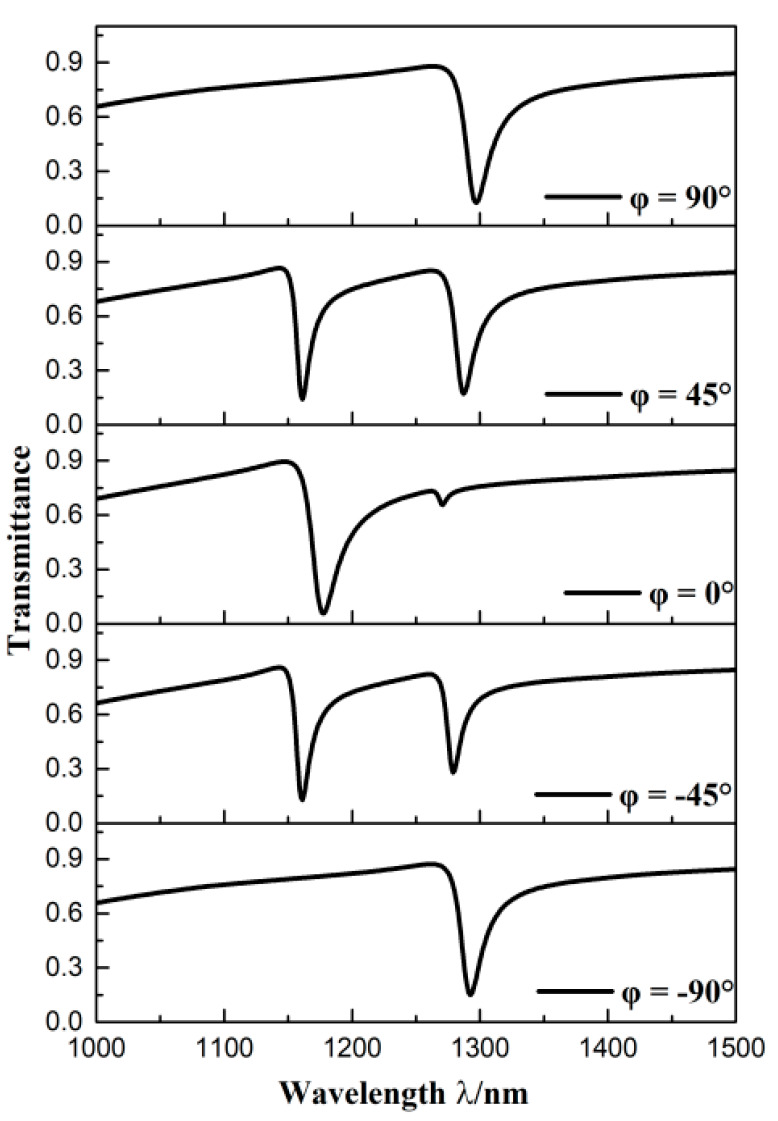
Transmission spectra of structures with diverse angle between horizontal axis and centerline of the stub on CRCS.

If the angle between the horizontal axis and centerline of stub on CRCS is different, the structure will vary, which could achieve diverse propagation characteristics. Hence, the structures with different *φ* set as 90°, 45°, 0°, −45°, −90° were investigated. As potted in Figure 3, the 90° structure and −90° structure had similar transmission spectra that had one dip with low transmittance. The 45° structure and −45° structure had similar transmission spectra that had two dips both with low transmittance. The transmission spectrum of 0° structure was different from others, which had two dips (one with low transmittance and another with high transmittance). This because that the 90° structure and −90° structure are symmetric about centerline, whereas the 45° structure, 0° structure, and 45° structure are asymmetric about the centerline. Besides, the symmetry degree of the 0° structure differs from that of the 45° structure and −45° structure. So, although these three structures all had two dips, the width and transmittance at dips on spectrum of the 0° structure were much different from that of the 45° structure and −45° structure. Noticeably, on the spectrum of the 0° structure, one dip had boarder width compared with other structures, another dip had high transmittance that is very close to the highest transmittance on the whole spectrum. It can be concluded that the transmission properties of the 0° structure are not as good as others. And the 90° structure and −90° structure, 45° structure and −45° structure have similar better transmission properties, respectively. Therefore, the detailed discussion of the 90° structure and 45° structure is in the following.

**Figure 4:**
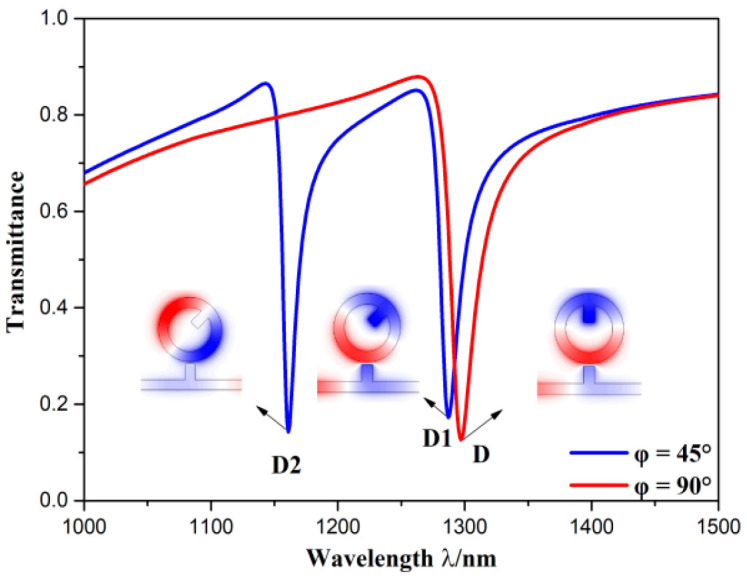
Transmission spectra of the 45° structure and 90° structure. The normalized H_Z_ field distributions at their dips are depicted in the inset.

The transmission spectra of the 90° structure and 45° structure are shown in Figure 4, and their normalized H_Z_ field distributions at dips are depicted in the inset. The two dips of the 45° structure are marked as D1 (*λ* = 1287 nm) and D2 (*λ* = 1161 nm), respectively. The dip of the 90° structure is denoted by D (*λ* = 1297 nm). It is observed that the spectral width at D1 and D2 was narrower than that at D. According to calculation, the FWHM at D1, D2, and D are 22, 14.6, 27.3 nm, respectively. Additionally, the normalized H_Z_ field distributions at these three dips all had two nodes, which indicates they are in the same order of mode of Fano resonance. However, their positions of nodes were diverse. Two nodes at D were symmetric about centerline, two nodes at D1 were symmetric about centerline of the stub on the CRCS, and two nodes at D2 were in the centerline of the stub on the CRCS. It can be inferred that geometric asymmetry induces the mode splitting of Fano resonance. Besides, because of the mode splitting, the FWHM at D1 and D2 became narrower than that at D, while the transmittance at D2 was similar to that at D and transmittance at D1 was slightly higher than that at D. Thus, mode splitting of Fano resonance can get better propagation characteristics.

**Figure 5:**
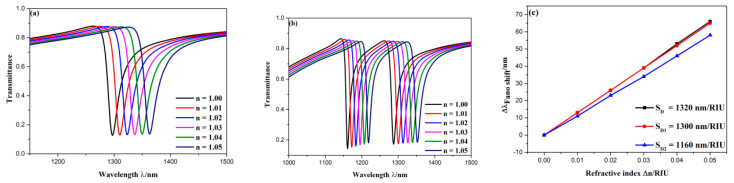
(**a**) Transmission spectra of 90° structure in diverse refractive-index; (**b**) transmission spectra of 45° structure in diverse refractive-index; (**c**) fitting line of sensitivity at D, D1, D2.

To further analyze the mode splitting of Fano resonance, the effect of the refractive index on transmission spectra of above two structures as well as their sensitivity were studied. The refractive index was varied from 1.00 to 1.05 RIU in the interval of 0.01 RIU. The influence of refractive indices on transmission spectra of the 90° structure and 45° structure are depicted in Figure 5a,b, respectively. It is seen that these two structures had similar change with the variation of refractive indices. To be specific, their spectra both showed a redshift with increase of refractive indices. And the refractive indices had less influence on their dip depth and spectral width. Moreover, as plotted in Figure 5c, the sensitivity at D, D1 and D2 all had excellent linear fitting, which is a significant factor to act as a sensor. The sensitivity of D, D1, D2 are 1320, 1300, 1160 nm/RIU, respectively. Then their FOM can be calculated, which are 48.35, 59.09, 79.45, respectively. It can be concluded that mode splitting of Fano resonance can vastly improve FOM with a little loss of sensitivity, and provide one more detection position. Therefore, the 45° structure is continued to discuss in the following, and the default structural sizes are the same as that of Figure 2.

**Figure 6:**
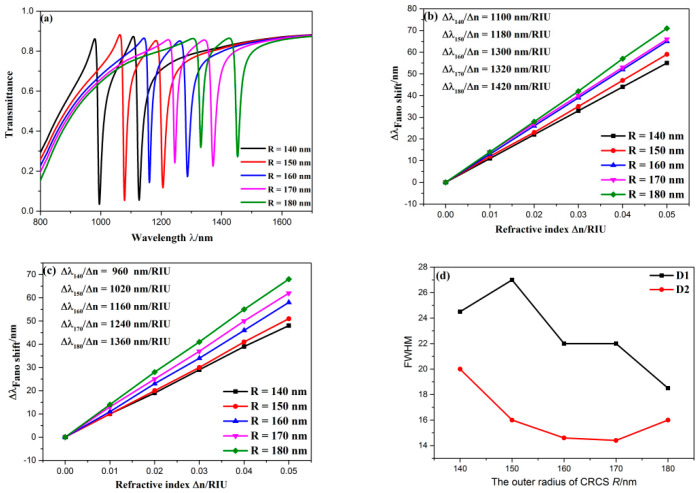
(**a**) Transmission spectra for diverse outer radius of CRCS; (**b**) fitting line of sensitivity at D1; (**c**) fitting line of sensitivity at D2; (**d**) the variation of FWHM with the rising of outer radius of CRCS.

First, the influence of outer radius of CRCS *R* was studied, which was gradually set as 140, 150, 160, 170, 180 nm. As plotted in Figure 6a, as *R* increased, the transmission spectra displayed an apparent redshift, the depth of two dips rose. As we analyzed before, the CRCS behaves as the narrowband mode that is an important factor for the formation of Fano resonance. Thus, when outer radius of the CRCS changes, the narrowband mode varies to induce the variation of position and depth at Fano resonance dip. The sensitivity change at D1 and D2 are depicted in Figure 6b,c, respectively. The sensitivity at D1 and D2 both steadily grew with the rising of *R*, and they both had great linear fitting. As shown in Figure 6d, the FWHM at D1 increased first then decreased with the rising of *R*. When *R* = 180 nm, the FWHM at D1 attained the smallest value (18.5 nm). Then, the FOM at D1 can be calculated, which is 76.76. Thus, when *R* = 180 nm, this structure achieved the best performance, which is 1420 nm/RIU with an FOM of 76.76. However, the FWHM at D2 decreased first then increased with the rising of *R*, and their turning points were different. When *R* = 170 nm, the FWHM at D2 obtained the smallest value (14.4 nm), and then the FOM at D2 is 86.11, which is the best FOM in this designed structure. Overall, larger *R* leads to better performance for sensing.

**Figure 7:**
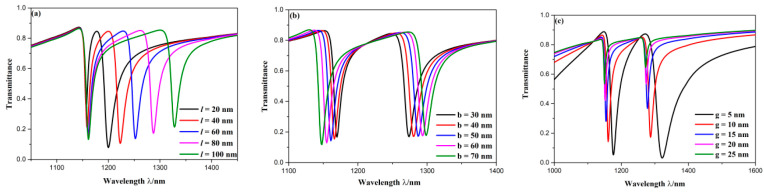
Transmission spectra for (**a**) diverse length of stub on CRCS; (**b**) diverse width of stub on CRCS; (**c**) diverse coupling gap.

The rest geometric parameters of CSCR are the length and width of stub on CRCS, which are denoted by *l* and *b*, respectively. The change of transmission spectra induced by *l* is depicted in Figure 7a, where *l* was set as 20, 40, 60, 80, 100 nm. As *l* increased, position of D1 moved to longer length and depth of D1 rose, however, position of D2 remained unchanged and depth of D2 dropped. Note that when *l* = 20 nm, the depth and width at D2 much differed from others. This is because 20 nm of *l* is very small, and then it slightly destroys symmetry, which leads to an incomplete mode splitting of Fano resonance. It is obvious that change of *l* had more influence on D1 rather than D2. This is because that the node position of D2 is in the centerline of stub on CRCS, while the antinode position of D1 is in the centerline of stub on CRCS, which is shown in Figure 4. No normalized H_Z_ field is at node, so the change of *l* has less influence on D2. The highest normalized H_Z_ field is at antinode, so the variation of *l* has strong effect on D1. The variation of transmission spectra induced by *b* is depicted in Figure 7b, in which *b* was set as 30, 40, 50, 60, 70 nm. As *b* grew, the whole spectral width and depth of D1 kept unchanged, the depth of D2 decreased, position of D1 showed a redshift but position of D2 emerged a blueshift. In other words, the distance between two dips became larger with the growth of *b*. It is also noticed from Figure 7a that the distance between two dips got larger with the rising of *l*. It indicates that the asymmetric degree of structures grows with the increase of *b* or *l*, which induces a larger degree of mode splitting of Fano resonance. However, the ways of effects of *b* and *l* on mode splitting of Fano resonance are diverse. Hence, the mode splitting of Fano resonance can be adjusted by changing *l* and *b*. In addition, based on the above analysis, it is found that structural parameters of CRCS have remarkably influence on positions of Fano resonance dips. It demonstrates that wavelengths of Fano resonance are mainly determined by narrowband mode.

The influence of coupling gap *g* was investigated, which is shown in Figure 7c. The *g* was gradually set as 5, 10, 15, 20, 25 nm. As *g* became larger, two dips showed an evident blueshift, transmittance at dips increased, the spectral width got narrower. This is because that as the coupling distance rises, coupling strength becomes weaker, which leads to the increase of transmittance at dips. Meanwhile, the coupling range gets narrower, which induces narrow spectral width. Therefore, it is necessary to choose better *g* based on the above analysis.

**Figure 8:**
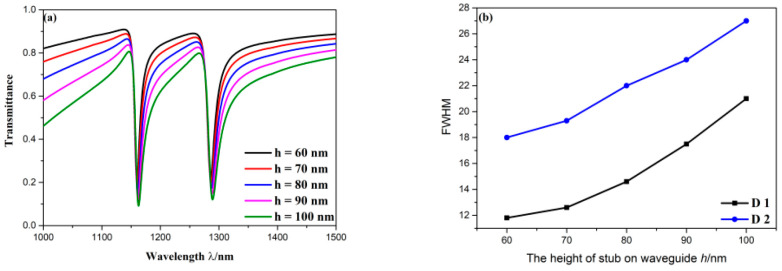

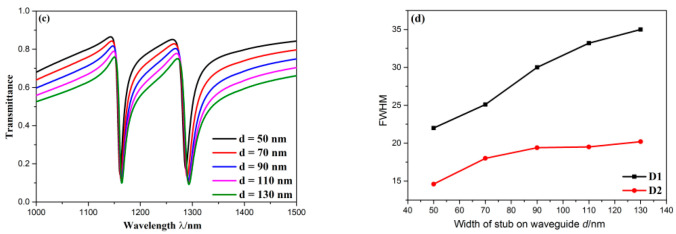
(**a**) Transmission spectra for diverse height of stub on bus waveguide; (**b**) the varying FWHM with rising of height of stub on bus waveguide; (**c**) transmission spectra for diverse width of stub on bus waveguide; (**d**) the varying FWHM with rising of width of stub on bus waveguide.

Successively, the structural parameters of stub on bus waveguide were investigated. The height of stub on bus waveguide *h* was set as 60, 70, 80, 90, 100 nm. It can be observed from Figure 8a that *h* had less influence on positions of dips but had remarkable effect on width of transmission spectra. Specifically, as *h* grew, the FWHM of D1 increased from 18 to 27 nm, the FWHM of D2 increased from 11.8 to 21 nm, which are shown in Figure 8b. The width of the stub on bus waveguide *d* was set as 50, 70, 90, 110, 130 nm. The variation of transmission spectra with change of *d* is depicted in Figure 8c. Similarly, when *d* grew, wavelengths of Fano resonance dips did not shift, the width of profile became broader. To be specific, as plotted in Figure 8d, the FWHM of D1 rose from 22 to 35 nm, the FWHM of D2 rose from 14.6 to 20.2 nm. In addition, transmittance at two dips slightly dropped with the growing of *h* and *d*. It can be concluded that smaller size of the stub on bus waveguide possesses narrower FWHM that leads to better optical resolution and FOM. Additionally, it can be found that the spectral width of Fano resonance largely depends on geometric parameters of the stub on bus waveguide, which acts as broadband mode. Furthermore, the stub on bus waveguide is always symmetric about centerline regardless of how *h* and *d* change. Therefore, the sizes of the stub on bus waveguide cannot affect the mode splitting of Fano resonance. Based on the above discussion, smaller *h* and *d* should be chosen in this structure.

## 4. The Application of the Presented Structure on Temperature Sensing

**Figure 9:**
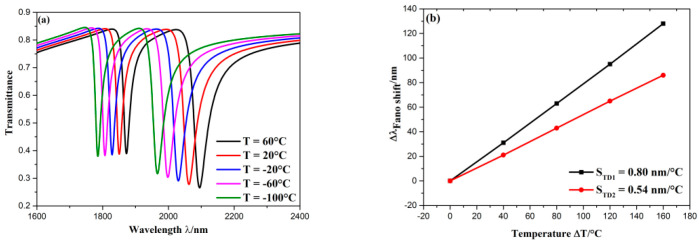
(**a**) Transmission spectra of diverse temperature; (**b**) the fitting line of sensitivity at D1 and D2.

The presented structure can also act as a temperature nanosensor. A temperature sensing material, ethanol, is filled in the bus waveguide and CRCS. The reason why we choose ethanol is that it characterizes the high refractive-index temperature coefficient (3.94 × 10^−4^), and there is a good linear relationship between the refractive-index and temperature, which is *n* = 1.36048 − 3.94 × 10^−4^ (*T* − *T*_0_). *T*_0_ is room temperature (initial temperature), which constantly equals to 20 °C; *T* is ambient temperature, which needs to be detected. The refractive-index temperature coefficient of Ag and quartz are 9.30 × 10^−6^ and 8.60 × 10^−6^, respectively, which are two orders of magnitude smaller than that of ethanol. Thus, the variation of temperature largely affects ethanol, and the effects of thermal expansion of Ag and quartz can be ignored. The sensitivity of temperature sensing can be expressed by S_T_ = ∆*λ*/∆*T*. The geometric parameters are set as follows: *R* = 180 nm, *b* = *d* = 50 nm, *l* = *h* = 80 nm, *φ* = 45°, *g* = 10 nm. The temperature was gradually set as 60, 20, −20, −60, −100 °C. As shown in Figure 9a, as temperature rose, the wavelengths of Fano resonance both showed an evident blueshift, the profile of Fano resonance kept unaltered. Since *T* is in an inverse ratio to *n*, the movement direction of Fano resonance dips when rising of temperature is opposite to that of refractive-index. The rest change of transmission spectra with the variation of temperature is similar to that of the refractive-index, which indicates the temperature sensor has similar properties as the refractive-index sensor. Moreover, the temperature sensitivity was measured, which is plotted in Figure 9b. It is seen that temperature sensitivity at two dips both had excellent linear fitting, and the sensitivity at D1 and D2 are 0.80 and 0.54 nm/°C, respectively.

## 5. Conclusions

In this work, a novel nanosensor structure that comprises an MIM bus waveguide with the stub and a circular ring cavity with a stub (CRCS) is proposed. The results reveal that the phenomenon of Fano resonance is emerged in the transmission spectrum, which is aroused from the interference between narrowband mode and broadband mode. The narrowband mode is determined by CRCS, which can influence the dips’ position of Fano resonance. The broadband mode relies on stub on bus waveguide, which can change FWHM of transmission spectra. Additionally, different structures with diverse angle between horizontal axis and centerline of stub on CRCS were investigated. It is found that symmetric structures had one Fano resonance dip, while asymmetric structures had two Fano resonance dips. It indicates that geometric asymmetry induces the mode splitting of Fano resonance. By comparing the 45° structure with 90° structure, it is observed that mode splitting of Fano resonance can hugely improve FOM with a little loss of sensitivity. The best performance is attained, which is 1420 nm/RIU with a high FOM of 76.76. Furthermore, the application of the presented structure on temperature sensing was discussed. The ethanol as temperature sensing material is filled in the CRCS and bus waveguide to realize better temperature sensing. The propagation characteristics of temperature nanosensor are similar to that of the refractive-index nanosensor, and its best sensitivity reaches 0.80 nm/°C. The designed structure also can potentially apply to other plasmonic optical devices.
